# Analysis of Cancer Genomic Amplifications Identifies Druggable Collateral Dependencies within the Amplicon

**DOI:** 10.3390/cancers15061636

**Published:** 2023-03-07

**Authors:** Guillem Pons, Gabriel Gallo-Oller, Natalia Navarro, Patricia Zarzosa, Júlia Sansa-Girona, Lia García-Gilabert, Ainara Magdaleno, Miguel F. Segura, Josep Sánchez de Toledo, Soledad Gallego, Lucas Moreno, Josep Roma

**Affiliations:** 1 Laboratory of Childhood Cancer and Blood Disorders, Vall d’Hebron Research Institute, Hospital Universitari Vall d’Hebron, Universitat Autònoma de Barcelona, 08035 Barcelona, Spain; 2 Pediatric Oncology and Hematology Department, Hospital Universitari Vall d’Hebron, Universitat Autònoma de Barcelona, 08035 Barcelona, Spain

**Keywords:** cancer, gene amplifications, CRISPR-Cas9 screenings, gene dependencies, drug development

## Abstract

**Simple Summary:**

Genomic amplifications are highly prevalent in cancer and often contribute to increased proliferation or cell survival upon the administration of anti-cancer drugs. The identification of those amplified genes at which cancer cells are selectively dependent is crucial for the development of new targeted therapies. On this matter, CRISPR/Cas9 screens have emerged as a useful tool to deplete the expression of almost all genes while assessing their consequences for cell survival. Here, we analyzed data from CRISPR/Cas9 screens in 954 cancer cell lines to identify selective gene dependencies associated with common cancer genomic amplifications. Our results suggest that cell lines of different tumor types harboring the same genomic amplification are dependent almost entirely on the same amplified genes, providing a set of new promising targets specific to each genomic amplification.

**Abstract:**

The identification of novel therapeutic targets for specific cancer molecular subtypes is crucial for the development of precision oncology. In the last few years, CRISPR/Cas9 screens have accelerated the discovery and validation of new targets associated with different tumor types, mutations, and fusions. However, there are still many cancer vulnerabilities associated with specific molecular features that remain to be explored. Here, we used data from CRISPR/Cas9 screens in 954 cancer cell lines to identify gene dependencies associated with 16 common cancer genomic amplifications. We found that high-copy-number genomic amplifications generate multiple collateral dependencies within the amplified region in most cases. Further, to prioritize candidate targets for each chromosomal region amplified, we integrated gene dependency parameters with both druggability data and subcellular location. Finally, analysis of the relationship between gene expression and gene dependency led to the identification of genes, the expression of which may constitute predictive biomarkers of dependency. In conclusion, our study provides a set of druggable targets specific for each amplification, opening the possibility to specifically target amplified tumors on this basis.

## 1. Introduction

Focal chromosomal amplifications often drive an increase in the number of copies of certain oncogenes in malignant tumors [1]. As a consequence, cancer cells often become “addicted” to the overexpressed oncogenes, increasing their malignancy and resistance to certain drugs [2]. Over the last 30 years, several studies have been conducted on assessing the effects of genomic amplifications on tumor prognosis. Overall, genomic amplifications such as *MYC*, *EGFR*, *CDK4*, or *ERBB2* have been correlated with worse survival rates in different tumors [3,4,5,6]. However, it should be noted that, regardless of whether genomic amplifications may be a factor of good or poor prognosis, only copy number in a few amplified genes has been established as a biomarker for targeted therapies (e.g., *ERBB2* amplification and trastuzumab sensitivity) [7]. Nevertheless, as we quantify copy-number variation for molecular diagnosis in a wide variety of tumors, the relevance in tumorigenesis of most genes embedded in the amplified regions remains unknown. Currently, this lack of knowledge hinders the clinical translationality of the molecular diagnosis towards the discovery and use of new targeted therapies for these patients. Thus, there is an unmet need to identify specific druggable dependencies associated with chromosome amplifications recurrently detected in tumors. On this matter, CRISPR/Cas9 dropout screens have emerged as a useful approach to discovering the role played by multiple proteins as well as identifying new targets for tumors with specific molecular features [8,9,10]. Meanwhile, some studies have highlighted the generation of false-positive hits in CRISPR screens in cancer cell lines harboring genomic amplifications, complicating the identification of copy-number-associated dependencies [11,12]. During the last few years, several methods, such as the Chronos algorithm, have been developed to address some CRISPR screen artifacts simultaneously, including the nonspecific CRISPR-cutting-induced toxicity observed in those sgRNAs targeting amplified regions [13,14]. Here, we used CRISPR screen data corrected by the Chronos algorithm to determine those genes on which cell lines harboring a high copy number of a specific amplification are dependent. We also integrated gene dependency data with druggability information to particularly prioritize actionable targets for each chromosomal amplification. Finally, RNA-seq gene expression data was used to identify those genes in which mRNA levels may constitute a predictive biomarker of response to their inhibition. Overall, our analysis provides insights into the importance of a wide range of amplified genes and identifies selective gene dependencies associated with each chromosomal amplification. These data suggest that these genes deserve to be further studied in preclinical or clinical settings and reinforce the idea of exploiting amplification-associated vulnerabilities to selectively target cancer cells.

## 2. Materials and Methods

### 2.1. Selection of Copy Number Amplifications in Tumors and Cell Lines

Copy-number data from 10,712 tumor samples were retrieved from a compilation of 32 TCGA Pan-Cancer Atlas Studies (RRID:SCR_014555) [15,16]. Copy-number data for 954 cancer cell lines were retrieved from the DepMap dataset Copy Number 21Q4 Public [17] (RRID:SCR_017655). Gene level copy number data available in DepMap were relative to each cell line ploidy and log2-transformed with a pseudo count of 1 (log2 relative copy number + 1). To establish which cell lines harbored an amplification in each gene analyzed, absolute copy numbers (ACN) were calculated from relative copy numbers (RCN) using the following formula: ACN = cell line ploidy × ((2^RCN) − 1). The following threshold was defined to consider high-copy number amplification: RCN ≥ 2, which is equal to an ACN ≥ 6 copies in diploid cells. For this study, 16 genomic amplifications were selected based on their frequency of occurrence in both TCGA tumor samples and DepMap cancer cell lines. This selection was necessary to ensure a sufficient number of amplified cell lines to conduct the analysis effectively. Then, well-characterized oncogenes that exhibit a high frequency of amplification were selected as reference genes for each amplification (Supplementary Table S1a). Coamplified genes were identified by correlating copy numbers of the reference genes with copy numbers of the remaining genes in cell lines. Genes whose copy numbers showed an *r* value ≥ 0.7 with the reference gene copy numbers were considered coamplified genes (Supplementary Table S1b). We also integrated data regarding chromosome location and gene type retrieved from BioMart [18] (RRID:SCR_002987) and Genecards [19,20] (RRID:SCR_002773). For reference genes located in adjacent chromosomal bands (e.g., *CDK4* and *MDM2*, located in chromosomes 12q14 and 12q15, respectively), a degree of amplification co-occurrence was detected and taken into account in further analysis.

### 2.2. Overall Survival and Disease-Specific Survival in Tumor Samples

Survival data from 10,712 tumor samples were retrieved from the TCGA Pan-Cancer Atlas Studies (RRID:SCR_014555). Median overall survival (OS), disease-specific survival (DSS), and hazard ratios (HR) were calculated to assess the effect of the 16 amplifications on patient prognosis. Samples bearing amplifications in the reference genes of each region were compared to samples with none of the amplifications. Stratification was applied to better understand the significance of each amplification within each tumor type. The significance between survival probability curves was determined using the log-rank test.

### 2.3. Comparison of Similarity in Genomic Amplification between Tumors and Cell Lines

To compare amplifications between tumors and cell lines, TCGA tumors (*n* = 10,712) and DepMap cell lines (*n* = 954) were grouped into 25 and 23 lineages, respectively, of which 21 were shared among them (Supplementary Tables S2a and S2b). Subsequently, the amplification frequencies in tumors and cell lines for each reference gene and lineage were correlated. A greater correlation between amplification frequencies in tumors and cell lines meant a greater similarity among them in terms of gene amplification. Therefore, in relation to the calculated Pearson’s correlation coefficient (*r*), three thresholds were established: high (*r* ≥ 0.7), moderate (0.7 < *r* ≥ 0.4), and weak or negligible correlation (*r* < 0.4).

### 2.4. Screening of Gene Dependencies Associated with Gene Amplifications

To analyze the dependencies associated with each gene amplification, gene effect data for 17,385 genes in 954 cell lines were retrieved from the DepMap dataset CRISPR 21Q4 Public+Score, Chronos [13,21,22] (RRID:SCR_017655). Gene effects are estimates (scores) that measure, in terms of cell survival, the size of the effect of knocking out a gene. A score of 0 is equivalent to a gene that is not essential; negative scores indicate that knocking it out will lead to decreased cell survival, whereas positive scores indicate increased cell survival. Subsequently, the differences in gene effects between those cell lines harboring each of the 16 amplifications (ACN ≥ 6 copies) with respect to those cell lines that did not harbor each of them (ACN < 6 copies) were calculated. The significance between gene effects was determined using a two-tailed Student’s *t*-test followed by a Benjamini-Hochberg correction to obtain FDR *q*-values. Then, for each amplification, we performed an overlap analysis between those significant dependent genes (query genes, (k)) and C1 positional gene sets (gene sets, (k)) using MSigDB [23] (RRID:SCR_016863). C1 positional gene sets are a compendium of 299 gene sets accounting for those human genes annotated on the GCh38.p13 reference chromosome bands. Thus, overlap analysis was performed to find chromosomal localization overlaps within those significant dependent genes. The ratio between the number of query genes (k) and the number of genes within each gene set (K) was plotted on the *x*-axis, whereas the gene set name was plotted on the *y*-axis. The FDR *q*-value was indicated by a color gradient, and the gene set size by a size dot gradient. Graphpad Prism 6.01 (RRID:SCR_017655) was used to generate the volcano plots and overlap plots. In addition, to confirm an enrichment of coamplified genes in the gene dependencies obtained, for each amplified region we ran a preranked GSEA [24] (RRID:SCR_003199), comparing the preranked list of dependent genes (ordered by *q*-value) with the corresponding gene set of coamplified genes. The following parameters were set: 10,000 permutations, a weighted enrichment statistic, and meandiv as the normalization mode. Finally, we used the normalized enrichment score (NES) and the associated FDR *q*-value to statistically interpret the enrichment plots obtained.

### 2.5. Prioritization of Candidate Targets for Each Chromosome Amplification

To prioritize candidate targets for each chromosome amplification, we integrated druggability and subcellular localization data from CanSAR [25] (RRID:SCR_006794) for the 923 significant gene dependencies identified (corresponding to 770 different genes) (Supplementary Table S3). Considering that cellular localization is important for target druggability, proteins secreted or located in the cell membrane were selected from those located in other cellular compartments. Then, structure-based ligandability scores, ranked from low ligandability (−3) to high ligandability (3), were added using a color gradient. To verify the results obtained in the initial pan-cancer screening, we also compared the difference in gene effects of those prioritized genes between amplified and non-amplified cell lines in selected lineages. In the case of *SLC26A10* and *CALM1P2*, as copy number data was not available, we used as a surrogate marker of amplification *B4GALNT1* and *EGFR*, respectively, which are genes closely located to both genes of interest. The significance between gene effects was determined using a two-tailed Student’s *t*-test. Graphpad Prism 6.01 (RRID:SCR_017655) was used to generate the plots.

### 2.6. Correlation between Gene Dependencies and Gene Expression

RNA-seq expression data for prioritized genes were retrieved from the DepMap dataset Expression 21Q4 Public [17] (RRID:SCR_017655). Gene expression data were log2 transformed with a pseudo-count of 1 (log2 TPM + 1). Since the immediate downstream effect of amplification is overexpression, we first determined which prioritized genes were overexpressed when amplified. To address that, for each prioritized gene, relative copy numbers were correlated with gene expression in selected tumor types. In the case of *SLC26A10*, we could not perform any correlation as neither copy number nor gene expression were available. In the case of *FKBP9* and *CALM1*, we correlated their expressions with *FKBP9P1* and *CALM1P2* copy numbers, respectively, as *FKBP9P1* and *CALM1P2* were found to be amplified but not *FKBP9* and *CALM1*. Then, to determine whether mRNA levels could be a predictive biomarker of gene dependency, gene expression was correlated with gene effect data in specific tumor categories because of putative lineage-specific differences in gene expression. The *p*-values obtained were corrected using the Benjamini-Hochberg correction to obtain FDR *q*-values. Graphpad Prism 6.01 (RRID:SCR_017655) was used to generate the plots.

## 3. Results

### 3.1. Cancer Cell Lines as a Model to Study Tumor Gene Amplifications

**Figure 1:**
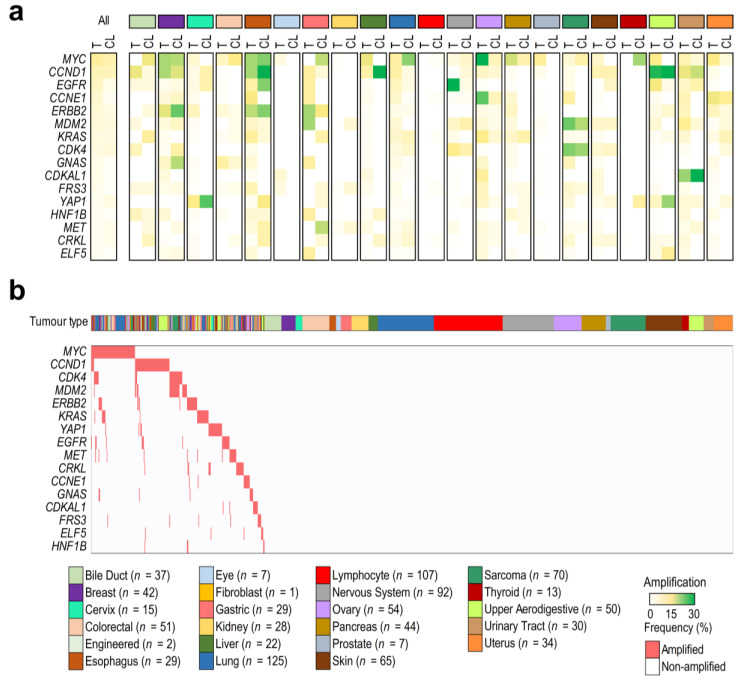
Cancer cell lines as models to study tumor gene amplifications. (**a**) Comparison of amplification frequencies between TCGA tumors (T) and DepMap cell lines (CL) from 21 lineages. A color gradient (from white to green) indicates the frequency of each amplification. (**b**) An amplification map of the DepMap cell lines (*n* = 954) used in the study. Cell lines were classified by the lineage of origin and ordered by the presence (red) or absence (white) of each amplification (≥6 copies).

Before gene dependency analysis, an initial key question was whether gene amplifications detected in cell lines reflected those observed in the tumors from which they were derived or whether particular amplifications had been selected in cell culture, implying an artifactual genetic bias. Correlations between gene amplification frequencies in tumors and cell lines showed that gene amplification profiles in cell lines resembled those observed in same-lineage tumors in most cases (Figure 1a). In particular, we found that amplification frequencies between tumors and cell lines showed a high correlation in 7/17 (41% of lineages) (breast, cervix, colorectal, ovary, sarcoma, upper aerodigestive, and urinary tract tumors), a moderate correlation in 6/17 (35% of lineages) (esophagus, liver, lung, pancreas, skin, and uterus), and a weak/negligible correlation in 4/17 (24% of lineages) (bile duct, gastric, nervous system, and thyroid) (Supplementary Figure S1a). It was not possible to correlate gene amplification frequencies in ocular, kidney, lymphoid, and prostate lineages since in these pathologies gene amplifications were found to be a rare phenomenon. To clearly visualize the data analyzed, a gene amplification map was generated for cell lines (Figure 1b) and tumors (Supplementary Figure S1b).

At this point, we also aimed to study the significance of each gene amplification in tumor survival by comparing amplified vs. non-amplified tumors. In the pan-cancer analysis, a decreased probability of overall survival (OS) and disease-specific survival (DSS) was found in amplified tumors in comparison with non-amplified ones (Supplementary Figure S2a,b), with *EGFR*, *CDK4*, *MDM2*, *KRAS*, and *CCNE1*-amplified tumors being those associated with a worse prognosis (Supplementary Figure S2c,d). Survival analysis by tumor type revealed that *EGFR* amplifications in low-grade gliomas and head and neck cancers, *CCNE1* amplifications in ovarian and uterine cancers, and *CDK4* amplifications in sarcomas were associated with a low probability of OS, suggesting that only certain amplifications in particular tumor types are relevant in prognosis (Supplementary Figure S2e–t).

### 3.2. Chromosome Amplifications Generate Collateral Dependencies within the Amplicon

**Figure 2:**
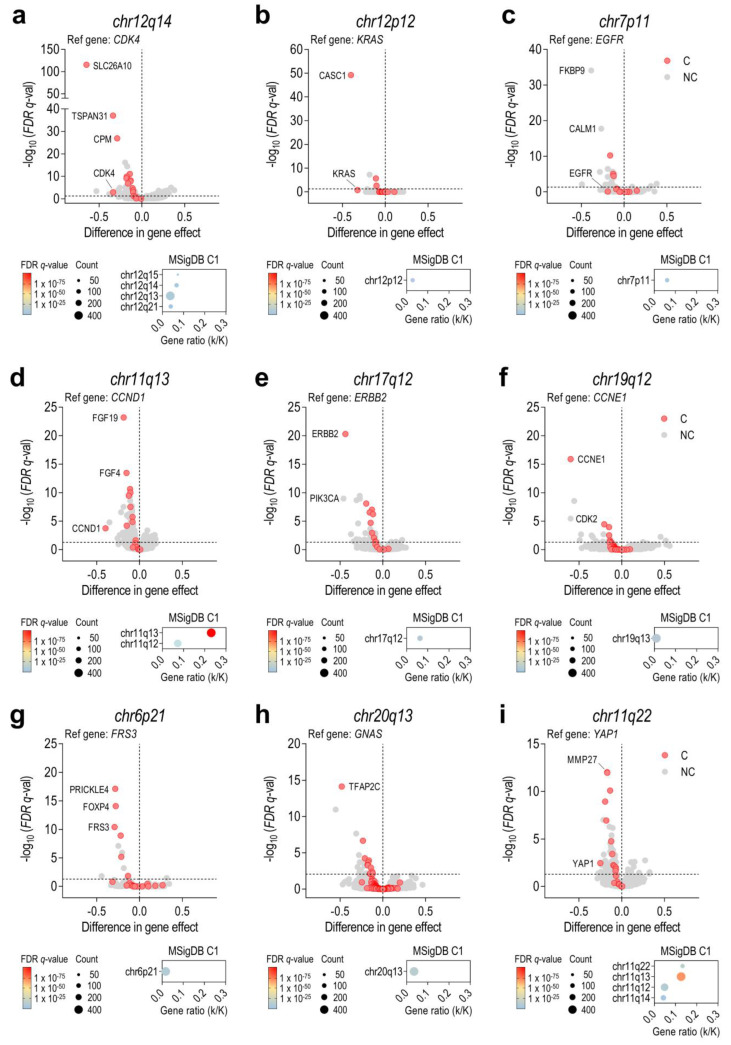
Identification of amplification-associated dependencies revealed the importance of coamplified genes. Results are shown for the following amplifications: (**a**) *CDK4*, chr12q14; (**b**) *KRAS*, chr12p12; (**c**) *EGFR*, chr7p11; (**d**) *CCND1*, chr11q13; (**e**) *ERBB2*, chr17q11; (**f**) *CCNE1*, chr19q12; (**g**) *FRS3*, chr6p21; (**h**) *GNAS*, chr20q13; and (**i**) *YAP1*, chr11q22. Top: Volcano plots showing the difference in the CRISPR-Cas9 gene effect between cell lines harboring amplifications (≥6 copies) or not (<6 copies). Genes were classified as coamplified (C, red dots) and non-coamplified (NC, grey dots). Statistical significance (*q* < 0.05) was determined using two-tailed *t*-tests followed by a Benjamini-Hochberg correction to obtain FDR *q*-values. Bottom: MSigDB overlap plots revealed an enrichment of amplification-associated dependencies in genes located within the same chromosomal band or in adjacent chromosomal regions. Gene ratio (k/K) refers to the overlap between the number of query genes (k) and the number of genes within each gene set (K). FDR *q*-value is indicated by a color gradient, and gene set size by a size dot gradient.

To elucidate the importance of each amplified gene in cell survival, all coamplified genes within each amplicon were identified. Then, for each amplicon, we found a distinct number and frequency of genes coamplified with the reference gene (Supplementary Figure S3a). The gene type (protein-coding genes, RNA genes, or pseudogenes) was considered since, for some non-coding genes, copy-number data were available but not CRISPR-Cas9 gene effect data. For this reason, we could not obtain relevant data for the *MYC* amplicon (chr8p24), since *MYC* and *POUF5F1B* were the only protein-coding genes coamplified in this region (Supplementary Figure S3b,c). In the remaining 15 regions, CRISPR-Cas9 gene effects were compared between amplified and non-amplified cells. Thus, we identified a set of genes whose disruption would impair the survival of cancer cells bearing each of the amplifications analyzed (Figure 2a–i and Supplementary Figure S3d–i). In addition, we used MSigDB to find overlapped chromosome localizations within those gene dependencies that were significant in the analysis (*q* < 0.05). Interestingly, most dependent genes were located within the same chromosomal band or, in some cases, in adjacent chromosomal regions (Figure 2a–i). A preranked GSEA was also performed to confirm an enrichment of coamplified genes among the most significant gene dependencies for each region analyzed (Supplementary Figure S4a–i). Of note, not all coamplified genes showed a collateral gene dependency, thus demonstrating the importance of finding those amplified genes with a higher influence on cell survival. In particular, *CDK4*-amplified cell lines were highly dependent on *SLC26A10*, *TSPAN31*, and *CPM*, all of which are within 12q13-q15 (Figure 2a). *MDM2*-amplified cell lines, which showed a high co-occurrence with *CDK4* amplification, showed a high dependency towards the same genes as *CDK4*-amplified cell lines and, in this case, also to *BEST3* (Supplementary Figure S3d). *KRAS*-amplified cell lines were especially dependent on *CASC1* (Figure 2b), and *EGFR*-amplified cell lines were highly dependent on *FKBP9* and *CALM1*. Intriguingly, neither *FKBP9* nor *CALM1* were located near or within the 7p11 region, but two of their pseudogenes, *FKBP9P1* and *CALM1P2*, were found to be coamplified with *EGFR* (Figure 2c). *CCND1*-amplified cell lines were especially vulnerable to *CCND1* depletion, but also to *FGF19* and *FGF4* depletion, while *ERBB2*-amplified and *CCNE1*-amplified cell lines strongly depended on *ERBB2* and *CCNE1*, respectively (Figure 2d–f). In the case of *FRS3*-amplified cell lines, a significant sensitivity to *PRICKLE4*, *FOXP4*, and *FRS3* depletion was observed, whereas *GNAS*-amplified ones strongly depended on *TFAP2C* and *YAP1*-amplified cell lines depended on *YAP1* or *MMP27* (Figure 2g–i). Additional relevant gene dependencies associated with amplifications in *CDKAL1*, *ELF5*, *CRKL*, *MET*, or *HNF1B* are shown in Supplementary Figure S3e–i.

### 3.3. Some Collateral Dependencies Generated by Amplification Are Druggable

**Figure 3:**
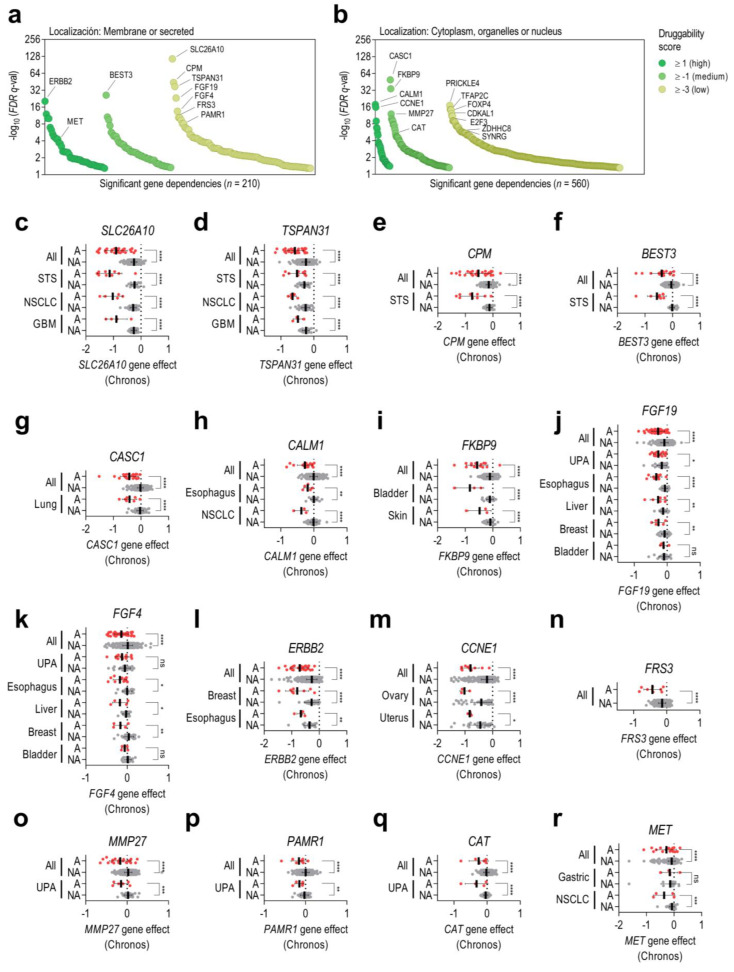
Prioritization of candidate targets for each chromosome amplification. CanSAR druggability scores (from +3 to −3) were integrated for all significant dependent genes previously identified. A higher druggability score indicates a higher probability of the codified protein harboring ligandable pockets within its 3D structure. Dependent genes were classified as (**a**) genes codifying for membrane or secreted proteins or (**b**) genes codifying for proteins located in the cytoplasm, organelles, or nucleus. (**c**–**r**) Differences in gene effects of the prioritized genes between amplified (A, red dots) and non-amplified (NA, grey dots) cell lines from specific tumor types. Results are shown for the following genes: (**c**) *SLC26A10*, (**d**) *TSPAN31*, (**e**) *CPM*, (**f**) *BEST3*, (**g**) *CASC1*, (**h**) *CALM1*, (**i**) *FKBP9*, (**j**) *FGF19*, (**k**) *FGF4*, (**l**) *ERBB2*, (**m**) *CCNE1*, (**n**) *FRS3*, (**o**) *MMP27*, (**p**) *PAMR1*, (**q**) *CAT*, and (**r**) *MET*. Statistical significance (*p* < 0.05) was determined using two-tailed *t*-tests. STS: soft-tissue sarcoma; NSCLC: non-small cell lung cancer; GBM: glioblastoma; UPA: upper aerodigestive tumors. * indicates *p* < 0.05; ** means *p* < 0.01, *** means *p* < 0.001, **** means *p* < 0.0001, and ns means non-significant.

Once gene dependencies associated with each gene amplification were characterized, we wondered which ones might be prioritized for their potential as putative new therapeutic targets. Prioritization for the significant gene dependencies previously found was based on both the subcellular localization and druggability score of each particular target (Figure 3a,b). Dependent genes were classified by the subcellular localization of the encoded protein, considering that membrane and secreted proteins are more accessible to being inhibited by small molecules or antibodies. Druggability scores were particularly taken into consideration for proteins located in the cytoplasm, nucleus, or organelles. Among dependent genes codifying proteins located in the membrane, we highlighted the following genes as priority targets: *SLC26A10*, *CPM*, *TSPAN31*, and *BEST3* (12q13–15), *FGF19* and *FGF4* (11q13), *ERBB2* (17q12), *FRS3* (6p21), *PAMR1* (11p13), and *MET* (7q31) (Figure 3a,b, Supplementary Table S3). Regarding dependent genes encoding proteins neither located in the membrane nor secreted, we prioritized *CASC1* (12p12), *CALM1* and *FKBP9* (*FKBP9P1* and *CALM1P2* pseudogenes located in 7p11), *CCNE1* (19q12), *MMP27* (11q12), and *CAT* (11p13) (Figure 3a,b, Supplementary Table S3). With the aim of confirming the results obtained in the initial pan-cancer screening, we also compared the difference in gene effects of the prioritized genes between amplified and non-amplified cell lines in specific tumor types (Figure 3c–r). Differences in gene effects between amplified versus non-amplified cell lines in distinct tumor types were similar, as it was observed in *SLC26A10*, *TSPAN31*, *CALM1*, *FKBP9*, *ERBB2*, or *CCNE1*, suggesting that the biological consequences derived from the knockout of these prioritized genes are, in general, lineage-independent. However, in some tumor types, no differences in gene effect were observed, as exemplified by *FGF19* and *FGF4* in bladder tumors or *MET* in gastric tumors.

### 3.4. mRNA Gene Expression Levels Only Correlate with Gene Dependency in Some Prioritized Genes

**Figure 4:**
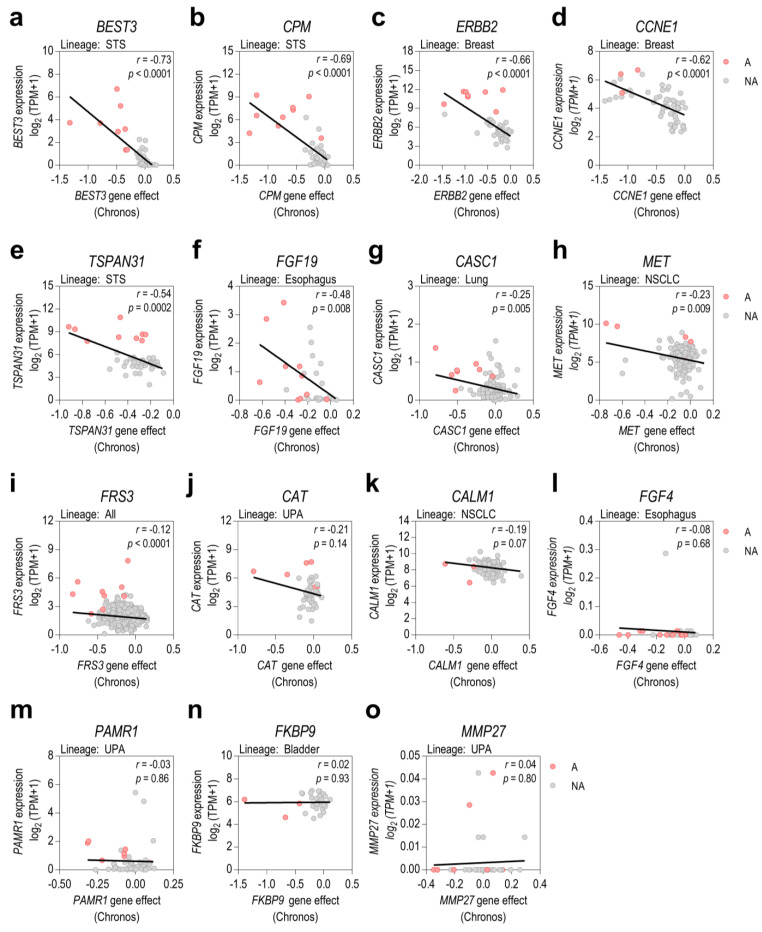
Assessment of gene expression as a predictive biomarker of gene dependency. (**a**–**o**) Pearson’s correlations between relative gene expression and gene knockout effects are shown for the prioritized genes in cell lines from selected lineages. Cell lines were also classified into amplified (A, red dots) and non-amplified (NA, grey dots). STS: soft-tissue sarcoma; NSCLC: non-small cell lung cancer; UPA: upper aerodigestive tumors.

Since the most plausible downstream effect of gene amplification is gene overexpression, we aimed to determine whether gene expression levels could be a predictive biomarker of gene dependency. To address this issue, we first determined which of the prioritized amplified genes were overexpressed when amplified in specific tumor categories. For each prioritized gene, we correlated its relative expression with its relative copy number (Supplementary Figure S5a–o). Our analysis showed that an increase in copy number does not always result in an increase in gene expression, potentially due to existing negative feedback mechanisms to regulate transcription or translation processes. Subsequently, relative expression was correlated with gene dependency data in cell lines from selected lineages (Figure 4a–o). Overall, the strongest correlations between expression and dependency were found in those prioritized genes whose copy number strongly correlated with gene expression (*BEST3*, *CPM*, *ERBB2*, *CCNE1*, or *TSPAN31*) (Figure 4a–e and Supplementary Figure S5a–e). However, in some cases, such as *CALM1*, *FGF4*, *FKBP9*, *PAMR1*, or *MMP27*, there was no clear correlation between gene amplification and gene overexpression, nor between gene expression and gene dependence (Figure 4k–o and Supplementary Figure S5k–o). Therefore, our results highlight, at least in the prioritized genes analyzed, the importance of taking gene copy number into account as a predictive biomarker of gene dependence instead of considering only mRNA levels.

## 4. Discussion

Over the last few years, several studies focused on assessing the effects of genomic amplifications on tumor prognosis have been conducted. However, only a few gene amplifications have been established as biomarkers for targeted therapies. In addition, the oncogenic relevance of most amplified genes remains unknown, thereby hindering the potential of molecular diagnosis towards the discovery and use of new targeted therapies to treat these tumors.

During the last few years, CRISPR-Cas9 loss-of-function screens have emerged as a powerful tool to identify essential gene dependencies for cancer cell line proliferation and survival while reducing the number of off-target effects that occur in RNAi screens [26]. However, CRISPR-Cas9 screens exhibit several biases and artifacts that may compromise the conclusions obtained. Recently, the newly developed algorithm Chronos has been shown to address various CRISPR screen artifacts simultaneously, thus exhibiting the lowest copy-number and screen quality bias of all evaluated methods [13]. In this study, we used Chronos-corrected data from CRISPR-Cas9 screens in 954 pan-cancer cell lines to reduce possible biases in the identification of gene dependencies associated with 16 common genomic amplifications detected in tumors.

Interestingly, correlations between gene amplification frequencies in tumors and cell lines showed that gene amplification profiles in cell lines resembled those observed in same-lineage tumors, enabling the translation of the findings in cell lines to human tumors (Figure 1a). Moreover, gene dependency analysis revealed a greater dependence among coamplified genes within each of the regions analyzed with respect to non-coamplified genes, thereby suggesting that some coamplified genes with previously unknown functions in tumor malignancy confer a higher survival capacity to cancer cells harboring each amplification (Figure 2a–i, Supplementary Figures S3d–i and S4a–i). In addition, we also integrated druggability data and subcellular localization to better select those gene dependencies that might be prioritized for further research and drug targeting (Figure 3a,b). Interestingly, differences in gene effects of prioritized genes between amplified and non-amplified cell lines were largely maintained when considering specific tumor types, suggesting that these amplification-associated dependencies are common among amplified cell lines independently of the tumor subtype (Figure 3c–r). These results are in concordance with the current knowledge that amplified tumors from distinct tumor types respond to targeted therapies directed at the amplification, as occurs with *ERBB2*/*HER2* or *MET*-amplified tumors [27,28]. Beyond the well-known *ERBB2*/*HER2* or *MET* amplification-associated dependencies (Figure 2e and Supplementary Figure S3h), there are other gene dependencies identified in the analysis that show promising results in selected tumor types. For example, inhibition of FGF19 in *CCND1*-amplified tumors (11q13 amplification) using anti-FGF19 antibodies has been shown to be an effective therapy for hepatocellular carcinoma (HCC), and the blockage of its FGFR receptors has shown promising clinical results in FGF19/FGF4+ HCC [29,30,31]. Other vulnerabilities, such as CDK2 and PKMYT1 dependencies in *CCNE1*-amplified tumors, are currently under study, and selective CDK2 inhibitors are starting to enter clinical development [32]. This part of the analysis provides a broad list of new potential druggable vulnerabilities associated with the most common amplifications detected in tumors, which may have a strong impact on the development of new targeted therapies. However, further in vitro/in vivo experimental validation will be required upon this initial screening.

Finally, another key point of our analyses was to determine whether mRNA levels could be used as predictive biomarkers of amplification-associated dependencies in all cases. It is well known that increases in gene copy numbers due to amplifications are often associated with concomitant increases in gene expression, which in turn can be observed in the mRNA levels of the amplified gene. However, there are several factors, such as negative feedback elements in transcriptional (or post-transcriptional) regulation, that may influence the mRNA levels of amplified genes. Interestingly, correlations between copy number and mRNA levels identified certain amplification-associated dependencies in which an increase in copy number did not result in a corresponding increase at mRNA level, such as *CALM1*, *FGF4*, *FKBP9*, *PAMR1*, or *MMP27* (Supplementary Figure S5k–o). For the particular case of these genes, mRNA may not be a reliable biomarker of dependence, and the focus should be on the copy number of the gene. These results indicate that some gene amplifications are not associated with increased mRNA levels despite showing a remarkable amplification-associated dependency. Conversely, for those genes whose copy number and mRNA levels correlate positively, such as *BEST3*, *CPM*, *ERBB2*, *CCNE1*, or *TSPAN31* (Figure 4a–e and Supplementary Figure S5a–e), both mRNA and copy number may be used as predictive biomarkers of their dependency. Further studies to assess protein levels would be necessary to better understand how the amplification of these genes contributes to their oncogenic potential.

We believe that these results could be reproduced and extended to almost all chromosomal amplifications detected in tumors, but a representative subset of cell lines harboring these particular amplifications would be required. Moreover, it would also be of utmost interest to analyze the effects derived from depleting ncRNA genes, especially those located in amplified regions that present a low frequency of protein-coding genes, such as the *MYC* amplicon, to obtain additional selective amplification-associated dependencies.

## 5. Conclusions

Our analyses identified new potential druggable vulnerabilities associated with recurrent chromosome amplifications detected in tumors. Some of these new amplification-associated dependencies included *SLC26A10*, *TSPAN31*, *CPM*, and *BEST3* for 12q13–15 amplifications; *CASC1* for 12p12 amplifications; *FKBP9* and *CALM1* for 7p11 amplifications; and *FRS3* for 6p21 amplifications, among others. In addition, our results supported previous findings on targeting amplification-associated dependencies in particular tumor types, such as *ERBB2*/*HER2* in 17q12 amplified breast cancer, FGF19/FGF4 in 11q13 amplified liver cancer, or *MET* in 7q31 amplified lung cancer, and suggested its extension to other tumors. Finally, gene expression analysis underscores the importance of considering copy numbers as a predictive biomarker of gene dependency instead of relying only on mRNA levels. In summary, we believe that the discovery of new vulnerabilities associated with recurrent amplifications detected in tumors might entail a major advance in the development of new therapies against cancer, thus contributing to the progress of precision medicine.

## Data Availability

The data generated in this study are available within the article and its supplementary data. All gene sets generated regarding the coamplified genes within each chromosomal region will be submitted for inclusion in MSigDB (RRID:SCR_016863), immediately after the acceptance of the manuscript. The data analyzed from TCGA (RRID:SCR_014555), DepMap (RRID:SCR_017655), CanSAR (RRID:SCR_006794), BioMart (RRID:SCR_002987), and Genecards (RRID:SCR_002773) were publicly available from their public websites, as stated in the corresponding section in Materials and Methods.

## Supplementary Materials

The following supporting information can be downloaded at: https://www.mdpi.com/article/10.3390/cancers15061636/s1. Figure S1: Amplifications in cancer cell lines and tumors; Figure S2: Survival associated with gene amplifications in TCGA tumors; Figure S3: Identification of amplification-associated dependencies reveals the importance of coamplified genes; Figure S4: Preranked GSEA reveals an enrichment in coamplified genes among significant dependent genes for each gene amplification; Figure S5: Copy numbers and gene expression correlations for the prioritized dependencies; Table S1a: List of co-amplified genes, reference genes, and the number of cancer cell lines showing each amplification; Table S1b: List of co-amplified genes with the reference gene for each amplification; Table S2a: Information and grouping of TCGA tumors; Table S2b: Information and grouping of DepMap cell lines; Table S3: Location and druggability scores for all dependent genes.
