# Supplementary material for: Analysis of Cancer Genomic Amplifications Identifies Druggable Collateral Dependencies within the Amplicon
Source: Cancers (Basel). 2023 Mar 7;15(6):1636. doi: 10.3390/cancers15061636 (PMC10046350; doi:10.3390/cancers15061636)
Supplement: Supplementary file 1 [file cancers-15-01636-s001.zip › Supplementary Figures.docx]

Communication

Analysis of Cancer Genomic Amplifications Identifies
Druggable Collateral Dependencies within the Amplicon

Guillem Pons ^1^, Gabriel Gallo-Oller ^1^, Natalia Navarro ^1^, Patricia Zarzosa ^1^, Júlia Sansa-Girona ^1^,
Lia García-Gilabert ^1^, Ainara Magdaleno ^1^, Miguel F. Segura ^1^, Josep Sánchez de Toledo ^1,2^, Soledad Gallego ^1,2^,
Lucas Moreno ^1,2^ and Josep Roma ^1,^*

**Supplementary Materials**

Figure S1: Amplifications in cancer cell lines and tumors; Figure S2: Survival associated with gene amplifications in TCGA tumors; Figure S3: Identification of amplification-associated dependencies reveals the importance of coamplified genes; Figure S4: Preranked GSEA reveals an enrichment in coamplified genes among significant dependent genes for each gene amplification; Figure S5: Copy numbers and gene expression correlations for the prioritized dependencies; Table S1a: List of co-amplified genes, reference genes, and the number of cancer cell lines showing each amplification; Table S1b: List of co-amplified genes with the reference gene for each amplification; Table S2a: Information and grouping of TCGA tumors; Table S2b: Information and grouping of DepMap cell lines; Table S3: Location and druggability scores for all dependent genes.

**

**

**

Supplementary Figure S1.** Amplifications in cancer cell lines and tumors. (**a**) Pearson’s correlations between amplification frequencies of TCGA tumors (T) and DepMap cancer cell lines (CL) from 21 lineages. (**b**) Amplification map of the TCGA tumors (n = 10712) used in the study. Tumors were classified by the lineage of origin and ordered by the presence (red) or absence (white) of each amplification.

**

**

**

**

**

**

**

**

**Supplementary Figure S2. Survival associated with gene amplifications in TCGA tumors**. (**a-b**) Kaplan-Meier curves showing the probability of patient's overall survival and disease-specific survival comparing those tumors which harbor any of the gene amplifications analyzed with those which did not harbor any of them. (**c-d**) Kaplan-Meier curves showing the probability of patient’s overall survival comparing those tumors which harbor each gene amplification with those which did not harbor any of them. (**e-t**) Kaplan-Meier curves showing the probability of patient’s overall survival comparing those tumors which harbor each gene amplification with those which did not harbor any of them in selected lineages where the amplification was frequently detected. Considering the number of patient samples harboring each amplification, 3 tumor types were selected regarding amplifications more frequently detected in TCGA tumors and 1 tumor type in amplifications less frequently detected. Statistical significance (*p* < 0.05) was determined using log-rank test.

**

**

**Supplementary Figure S3.** Identification of amplification-associated dependencies reveals the importance of coamplified genes. (**a**) Left: Number of coamplified genes with the reference gene. Right: Frequency of coamplified genes by its correlation coefficient with the reference gene. Below: Frequency of coamplified genes by its gene type (protein coding, lncRNA, miRNA, snoRNA or pseudogenes). (**b**) Volcano plots showing the difference in gene effect between cell lines harboring MYC amplifications or not. (**c**) The majority of coamplified genes with MYC (*r* > 0,7) are non-coding genes. (**d-i**) Volcano plots showing the difference in gene effect between cell lines harboring the remaining 5 amplifications analyzed: (**d**) *MDM2*, chr12q15 (**e**) *CDKAL1*, chr6p22 (**f**) *ELF5*, chr11p13 (**g**) *CRKL*, chr22q11 (**h**) *MET*, chr7q31 (**i**) *HNF1B*, chr17q12. Statistical significance (*q* < 0.05) was determined using two-tailed t-tests followed by Benjamini-Hochberg correction to obtain FDR *q*-values.

**

**

**Supplementary Figure S4.** Preranked GSEA reveals an enrichment in coamplified genes among significant dependent genes for each gene amplification. Enrichment plots are shown for 9 of 16 amplifications analyzed: (**a**) *CDK4*, chr12q14 (**b**) *KRAS*, chr12p12 (**c**) *EGFR*, chr7p11 (**d**) *CCND1*, chr11q13 (**e**) *ERBB2*, chr17q11 (**f**) *CCNE1*, chr19q12 (**g**) *FRS3*, chr6p21 (**h**) *GNAS*, chr20q13 (**i**) *YAP1*, chr11q22. Normalized enrichment scores (NES) were used as a primary statistic to compare gene set enrichment results and the associated *p*-value were used to assess the statistical significance (*p* < 0.05).

**

**

**Supplementary Figure S5.** Copy number and gene expression correlations for the prioritized dependencies. (**a-o**) Pearson’s correlations between gene relative copy number and relative gene expression are shown for those prioritized genes in cell lines from selected lineages. STS: soft-tissue sarcoma; NSCLC: non-small cell lung cancer; GBM: glioblastoma, UPA: upper aerodigestive tumors.
